# *Staphylococcus aureus* colonization in hemodialysis patients: a prospective 25 months observational study

**DOI:** 10.1186/s12882-019-1332-z

**Published:** 2019-05-06

**Authors:** Matthias Scheuch, Sabrina Freiin von Rheinbaben, Antje Kabisch, Jonas Engeßer, Susanne Ahrendt, Thomas Dabers, Christian Kohler, Silva Holtfreter, Barbara M. Bröker, Sylvia Stracke

**Affiliations:** 1 Department of Internal Medicine A, Division of Nephrology, University Medicine Greifswald, Greifswald, Germany; 2 Kuratorium für Dialyse und Nierentransplantation e.V., KfH-Nierenzentrum Greifswald, Greifswald, Germany; 3 Institute of Medical Microbiology, University Medicine Greifswald, Greifswald, Germany; 4 Institute of Immunology and Transfusion Medicine, University Medicine Greifswald, Greifswald, Germany

**Keywords:** *S. aureus*, Renal, Hemodialysis, AV fistula, CVC, Carrier, *spa* typing, Clonal complex, MLST, Mortality

## Abstract

**Background:**

Dialysis patients are frequently exposed to *Staphylococcus aureus* due to stays in dialysis centers, hospitals or rest homes. The hemodialysis vascular access is a potential entry site for *S. aureus*, in particular when using a central venous catheter (CVC) which increases the risk of sepsis compared to arteriovenous (AV) fistula. We prospectively followed a cohort of 86 hemodialysis patients from an outpatient dialysis center over 25 months analyzing *S. aureus* carrier status, *S. aureus* infection rates and mortality.

**Methods:**

Demographic data and patients´ medical histories were collected and followed from all hemodialysis patients. Blood samples, nasal swabs and swabs from the hemodialysis vascular access site were taken every six months for a period of 25 months and tested for *S. aureus*. Strains were cultured and further characterized by *spa* PCR and microarray-based genotyping. Resulting data were compared with those from the general population.

**Results:**

In cross-sectional analyses, an average of 40% of hemodialysis patients were *S. aureus* carriers compared to 27% in the general population. Longitudinally, a total of 65% were *S. aureus* carriers: 16% were persistent carriers, 43% were intermittently colonized. The most common *S. aureus* lineage in the dialysis patient cohort was the clonal complex (CC) 8 and the *spa* type t008, while in the general population, the clonal complex CC30 dominates. During the study period, we observed six *S. aureus*-associated blood stream infections with one *S. aureus* attributable death. *S. aureus* carriers with an AV fistula were more densely colonized in the nasal mucosa compared to patients with a CVC. Overall mortality was lower for hemodialysis patients with a positive *S. aureus* carrier status compared to non-carriers (hazard ratio of 0.19).

**Conclusions:**

Compared to the general population, hemodialysis patients were more frequently colonized with *S. aureus* and displayed both different *S. aureus* colonization densities as well as lineages, possibly explained by more frequent exposure to health care environments. The lower overall mortality in carriers compared to non-carriers is intriguing and will be investigated in detail in the future.

**Trial registration:**

ISRCTN 14385893, 2. October 2018, retrospectively registered.

**Electronic supplementary material:**

The online version of this article (10.1186/s12882-019-1332-z) contains supplementary material, which is available to authorized users.

## Background

The bacterium *Staphylococcus aureus* (*S. aureus*) is a ubiquitous microorganism in both healthy and immunocompromised hosts. The primary niche of these bacteria is the anterior nares [1]. However, they can also colonize other areas of the human body as part of the normal microbiota of the skin and the mucosa. Heijer et al. studied the prevalence of nasal *S. aureus* carriage across nine European countries and reported an overall crude prevalence of 21.6% (range 17–25%) considering 6956 patients seen by general practitioners [2]. The Study of Health in Pomerania (SHIP-TREND-0) revealed a prevalence of 27% *S. aureus* carriers in a general adult population in North East Germany [3]. Longitudinal studies revealed three patterns of *S. aureus* nasal carriage. Approximately 20% (range 12–30%) of healthy adults are persistent carriers and colonized by a single strain. 30% (range 16–70%) are intermittent carriers harboring different strains while 50% (range 16–68%) are persistent non-carriers [4]. The percentages can vary greatly due to use of different culture techniques, populations being studied, considered periods of time and interpretation guidelines [4].

*S. aureus* can either act as a harmless, persistent companion in the nose but may also lead to life-threatening infections if the host is immunocompromised or physiological barriers are breached, such as in hemodialysis patients [5, 6]. In 80% of *S. aureus* bacteremia cases, the infection has an endogenous origin, as shown by genotyping invasive and colonizing *S. aureus* isolates [7]. *S. aureus* infections are associated with high morbidity and mortality. A contributing factor is likely the ability of *S. aureus* to persist intracellularly or in biofilms, where they are protected from the host’s immune response and antimicrobial therapies. Biofilms are defined as a structural community of staphylococci enclosed in a self-produced slimy matrix that is attached to host tissue or implanted materials (e.g. catheters, and prosthetics). *S. aureus* infections are highly diverse, ranging from local infections, such as abscesses, to severe systemic infections, such as sepsis.

Dialysis patients are frequently exposed to *S. aureus* due to their regular stays in dialysis centers, hospitals or rest homes. Several studies reported an elevated *S. aureus* carriage rate of 51% in hemodialysis and 43% in CAPD patients while considering a percentage of 37% in the normal population [8].

Hemodialysis patients need a vascular access for dialysis – ideally an arteriovenous (AV) fistula, but in up to 34% of cases, a permanent central venous catheter (CVC) is used [9]. Regardless of the access type, these patients are more susceptible to bacterial infections and can subsequently develop bacteremia or sepsis with *S. aureus* as the main cause [10]. In detail, *S. aureus* carriers on hemodialysis have a 1.8 to 4.7-fold increased likelihood of suffering from a bacteremia compared to non-carriers on hemodialysis [4] - mostly as an endogenous infection. At the same time, there is a 20-fold increase in mortality in hemodialysis patients in the case of sepsis as compared to mortality data due to sepsis in general population [11].

The clinical situation is made more difficult by the spread of methicillin-resistant *S. aureus* strains (MRSA) [12]. In Germany, MRSA is responsible for 2% of nosocomial infections [13]. Particularly health care-associated MRSA (HA-MRSA) are among the most common pathogens of treatment-associated infections in medical and nursing facilities [14]. In addition to health care facilities, so called community-associated MRSA (CA-MRSA) can be acquired by traveling to high-prevalence areas and household contact with persons that harbor MRSA. These factors increase the colonization probability by MRSA and complicate both the procedures during dialysis and therapy in case of a systemic infection.

In a prospective 25 months observational study carried out in an outpatient dialysis center, we wanted to (1) determine the proportion of patients who are persistent or intermittent methicillin-sensitive *S. aureus* (MSSA) or MRSA carriers, (2) compare *S. aureus* subtypes in hemodialysis patients with data from general population of the same region (SHIP-TREND-0), (3) find and follow *S. aureus* infections in individual patients and (4) identify *S. aureus-*related factors contributing to overall mortality in this hemodialysis population.

## Methods

### Study cohort

66 subjects from an outpatient dialysis center (KfH e.V.) in Greifswald were included in the prospective SaDial-study (*Staphylococcus aureus* in dialysis patients) in February 2016. During the sampling period, another 20 patients were recruited, resulting in a total of 86 participants. All patients completed a questionnaire regarding their sociodemographic data and their medical history. Swabs were taken from the nasal mucosa and also from the surrounding skin at the catheter entry site, if present. This sampling was repeated for all dialysis patients included in August 2016, February 2017, September 2017 and in February 2018. 22 participants died during follow-up. Causes of death were taken from the final medical reports. Four of the authors (S.R., S.A., T.D., S.S.) are the nephrologists in charge for the patients reported here. All information contained in the medical charts were evaluated again and summarized for the study.

### Nose and catheter swabs

Swabs were obtained from two trained and validated investigators from the nasal mucosa and the surrounding skin at the catheter entry site by using BBL™ CultureSwabs from BD. In nasal swabs, the cotton tip was inserted about 1 cm into one nostril and rotated 4 times along the nasal septum with slight pressure against it and with slight rotational movements. The same procedure was repeated with the same swab in the other nostril. For sampling at catheter entry sites, the swab end was drizzled with a sterile physiological saline solution and rotated on the skin around the catheter entry with slight pressure. Subsequently, the swab samples were tested for *S. aureus* using a semiquantitative procedure. *S. aureus* enrichment took place by a phenol red mannitol salt broth and quantification on mannitol salt agar (BD, Heidelberg, Germany) as previously reported [3]. CFUs were determined and categorized as < 10, 10–10^2^, 10^2^–10^3^ and > 10^3^. From each *S. aureus*-positive patient sample, a single colony was picked for preparing the glycerol stock. DNA isolation was carried out using the DNeasy® Blood & Tissue Kit (250) from QUIAGEN (Qiagen, Venlo, The Netherlands) according to the manufacturer’s instructions, but with an addition of 0.2 mg/mL lysostaphin (Sigma-Aldrich, St. Louis, Missouri, USA) to the lysis buffer.

### Spa typing

The *spa* region refers to a repeat region on the protein A gene. The repeats usually have a length of 24 base pairs and vary in number from 2 to 16. Based on order and type of repeats, *S. aureus* strains are classified by the Ridom software and entered into the Ridom SpaServer database ([15], Ridom SpaServer (http://www.spaserver.ridom.de/). The amplification of the *spa* sequence was based on the protocol of ridom BIOINFORMATICS using the corresponding primers (http://www.ridom.de/staphtype/spa_sequencing.shtml). The initial temperature for denaturation of the strands was raised from 80 °C to 94 °C and the number of cycles decreased from 35 to 30. Subsequently, 10 μl of amplified spa DNA were applied to a 1.5% agarose gel for control for unspecific bands. 5 μl of amplified *spa* DNA per sample were incubated with 2 μl of ExoSAP-IT® from Affymetrix for 15 min at 37 °C and for further 15 min at 80 °C. The amount of purified PCR product was then determined by the DS-11+ Spectrophotometer from DeNovix and checked qualitatively. Based on the shown fragment length on the gel, the purified PCR product was diluted to 1 ng/μl (fragments to 300 bp) or 5 ng/μl (fragments from 300 bp) according to the protocol provided by eurofins. Each 15 μl of this dilution was separately combined with 2 μl (10 μM) of the spa-forward primer and the spa-reverse primer and sent to eurofins for sequencing. High-quality sequences were then integrated into the Ridom SpaServer database and assigned to a specific *spa* type.

### DNA microarray

The genetic composition of the obtained *S. aureus* isolates was determined with the *S. aureus* Genotyping Kit 2.0 from Alere Technologies GmbH [16]. This array contains single strand DNA probes for various virulence and resistance genes. Hybridization with biotin-labelled bacterial DNA allows the simultaneous detection of numerous staphylococcal genes. PCR for the linear amplification, biotin labeling of the nucleotide sequences and the subsequent hybridization of the PCR product on the chip surface were performed using the protocol provided. Bound bacterial DNA was visualized by precipitation staining and analyzed using the ArrayMate Reader from Alere Technologies GmbH. The result files include a qualitative statement about the gene expression (positive/negative/ambiguous) as well as signal intensities of all sequences spotted on the chip. Since each lineage is defined by its core variable genome and linked to defined mobile genetic elements, the virulence and resistance gene patterns detected by the Staph array can be used to predict the lineage type. Hence, the software automatically predicts the CC and provides a list of common multi-locus sequence typing (MLST) and *spa* types [16].

### Statistical analyses

Sociodemographic analyses were performed in Microsoft Excel 2010 (version: 14.0.7194.5000 (32-bit),© 2010 Microsoft Corporation) using the chi-square test for independence of two statistical characteristics. Multivariate survival analyses were performed using cox regression in SPSS (IBM© SPSS© Statistics, version 25) including factors such as age, duration of dialysis (years), gender (male/female), dialysis access (CVC/ AV fistula), *S. aureus* carrier status (carrier/non-carrier), BMI, diabetes status (diabetic/non diabetic), presence of foreign body material (CVC, implants), previous ICU stays, living situation (home/rest home, assisted living), (former) alcohol consumption, smoking behavior (smokers, ex-smokers/non-smokers) and previous bloodstream infection. For factors with significant impact on overall mortality, the hazard ratio (HR) is shown. Subsequently, these factors were tested for possible confounders or competing events by adding further factors cumulated and not cumulated. Factors leading to changes within the model coefficients by > 10% were considered as confounders. For all statistical analyses, a confidence interval of 95% was chosen.

## Results

### Patient characteristics

In total, 86 patients from an outpatient dialysis center were included in the SaDial-study. 70% were male; the median age was 65 years and 59% were older than 60 years (Table 1). 38% were dialyzed at least temporarily respectively at the time of sampling via CVC. 40% of the patients were diabetics and 62% stayed at least once on an intensive care unit (ICU). Only 5% were living at a nursing home. Almost half of the patients were dialyzed for more than 5 years. During follow-up time, 22 patients died, 7 withdrew from the study for other reasons, 4 patients were transplanted, 2 moved away and in further 3 patients, a dialysis was no longer necessary.

**Table 1:** Descriptive characteristics of the SaDial cohort (86 patients)

Variable	Amount	Portion (%)
Sex		
Female	26	30.2
Male	60	69.8
Age (yr)		
20–29	3	3.5
30–39	3	3.5
40–49	4	4.7
50–59	25	29.1
60–69	14	16.3
70–79	11	12.8
80–92	26	30.2
BMI		
< 18.5	2	2.3
18.5–25	30	34.9
25–30	28	32.6
30–35	21	24.4
35–40	4	4.7
> 40	1	1.2
Years requiring dialysis		
2–4	48	55.8
5–7	20	23.3
8–10	9	10.5
11–19	9	10.5
Housing situation		
House/flat with family	81	94.2
Nursing home/assisted living	5	5.8
Dialysis access		
AV Fistula	53	61.6
CVC	33	38.4
S. aureus carriage		
Yes	50	58.1
No	36	41.9
Diabetic		
Yes	34	39.5
No	52	60.5
Bloodstream infection		
Yes	17	19.8
No	69	80.2
Smoker		
Yes	12	14.0
Former	35	40.7
No	39	45.3
Alcohol consumption		
Yes	5	5.8
Former	37	43.0
No	44	51.2
Ever stayed on ICU		
Yes	53	61.6
No	33	38.4
Deceased during the course of the SaDial-study		
Yes	22	25.6
No	64	74.4

### *S. aureus* colonization and carrier status

**Fig. 1:**
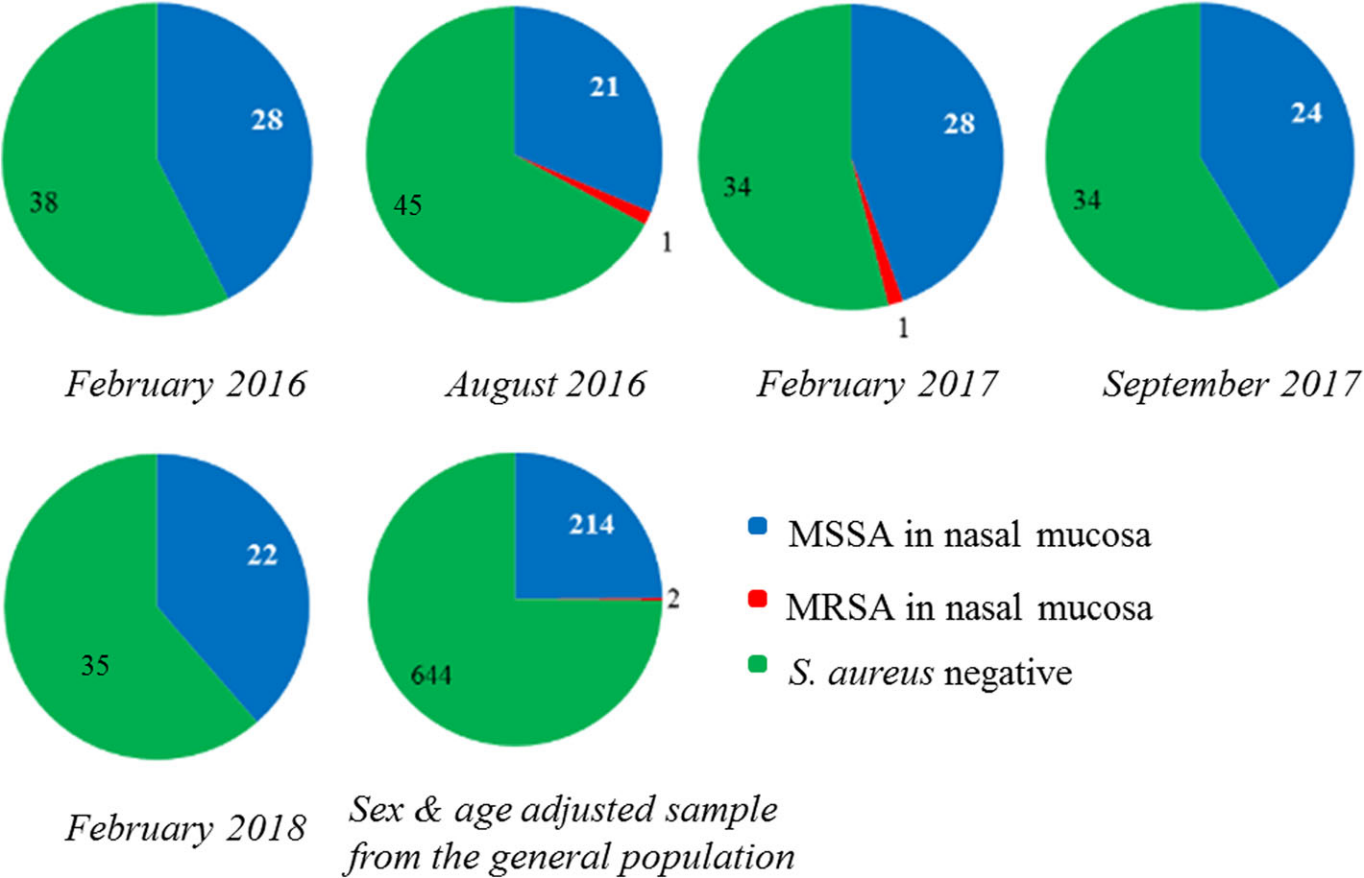
Methicillin-sensitive *Staphylococcus aureus* (MSSA) and Methicillin-resistant *Staphylococcus aureus* (MRSA) prevalence in nasal swabs from 86 dialysis patients from an outpatient dialysis center during biannual sampling from February 2016 to February 2018 and from sex- and age-adjusted sample from the general population. Cross-sectional carriage varied between 33 and 46%

During the study period, the colonization status of the dialysis patients was evaluated five times, every six months. The *S. aureus* carriage rate in the nasal mucosa varied cross-sectionally between 33 and 46%, depending on the sampling month (Fig. 1). Related to patients dialyzed via AF, the carrier rate was at 0.39, 0.46 for patients with a CVC access respectively.

In only five cases and three different patients, the bacterium could be detected at the catheter entry site (Additional file 1, ID 003, 005, 022). In February 2016, the *S. aureus* isolates from nose and CVC were identical in two cases. In the other three cases (two in February 2017, one in September 2017) the strains were unrelated.

Among all *S. aureus*-positive samples, only two MRSA were detected (ID 001, 072). The first patient died shortly before the following sampling took place due to respiratory failure with positive detection of *P. aeruginosa* in sputum. The second patient died three months after sampling due to a septic shock with unclear focus but possible CVC sepsis without pathogen detection.

**Fig. 2:**
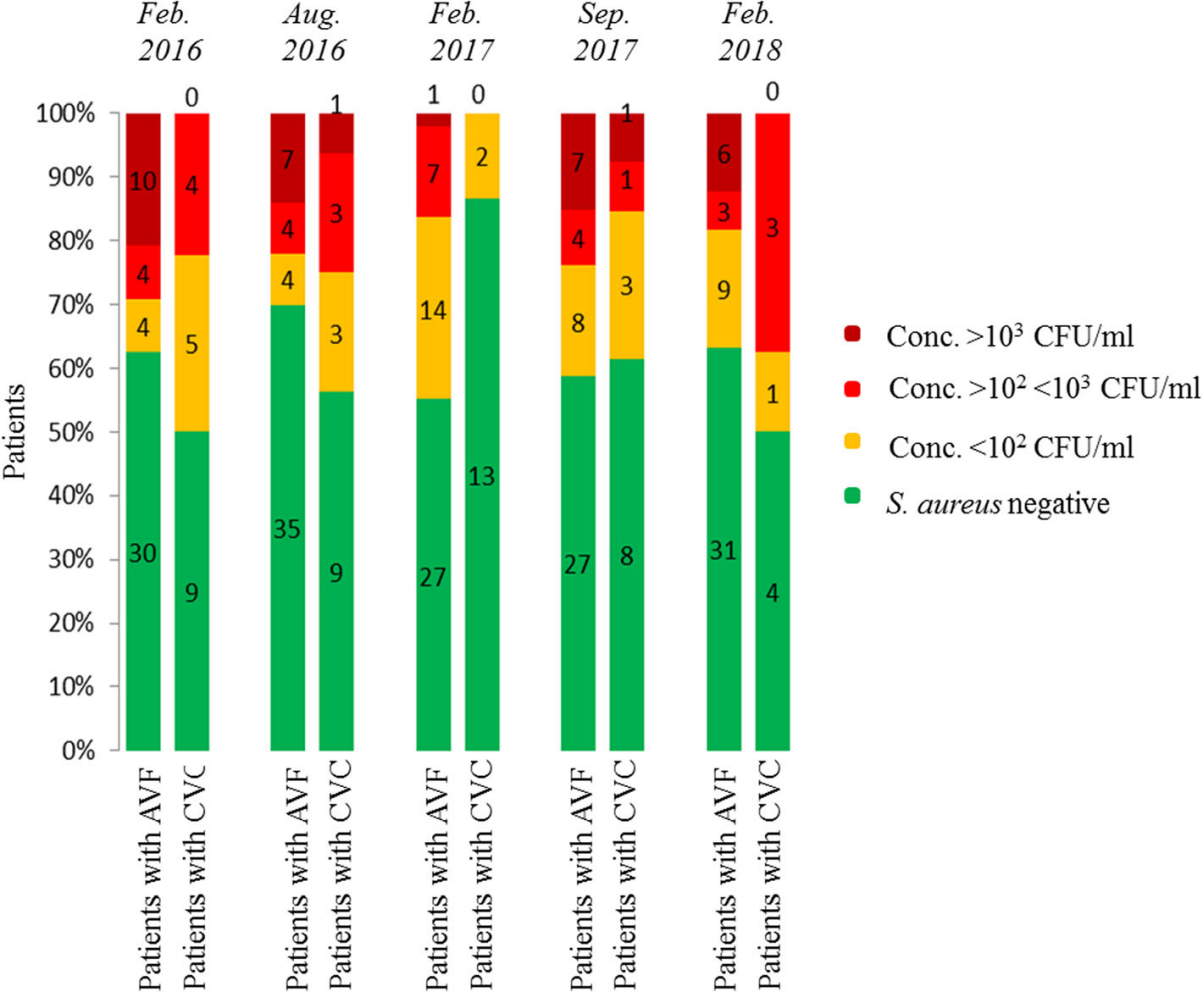
Nasal colonization density in patients dialyzed by an arteriovenous fistula (AVF) or a central venous catheter (CVC) from February 2016 to February 2018. About 1/3 of the cohort was dialyzed at least temporarily by a CVC. The number of colony forming units per milliliter (CFU/ml) is higher in patients with an AVF compared to the CVC cohort

Investigation of nasal colonization density revealed differences in the detected amount of colony forming units per milliliter (CFU/ml) of *S. aureus* in the nasal mucosa depending on the vascular access type (Fig. 2). While in CVC patients only in two cases a concentration of more than 1000 CFU/ml was detected, patients dialyzing via AV fistula were generally more densely populated with *S. aureus* (*p*-value = 0.045). In those five cases in which the bacterium was detected at the catheter entry site, the amount of CFUs was generally in the range of the amount of colony forming units detected in the nasal mucosa of the individual patient.

Longitudinally, 65% of hemodialysis patients showed a *S. aureus* colonization. A closer look at the population dynamics of the nasal colonization revealed that 14/86 patients (16%) were persistent *S. aureus* carriers, defined as being positive in at least 75% of samplings (Additional file 1). In 5 of these 14 patients the same strain was detected in the nasal mucosa during regular sampling. In the remaining 9 patients, the *S. aureus* strain changed to another unrelated strain. Another 37/86 (43%) patients were intermittent carriers with only one or two *S. aureus*-positive nose swabs during regular sampling. 5/86 patients (6%) could only be sampled once due to withdrawals, deaths or later study participation but showed a *S. aureus* colonization. The remaining 30/86 patients (35%) had no nasal *S. aureus* colonization and were thus classified as non-carriers.

### Characterization of the *S. aureus* strains and comparison with general population

**Fig. 3:**
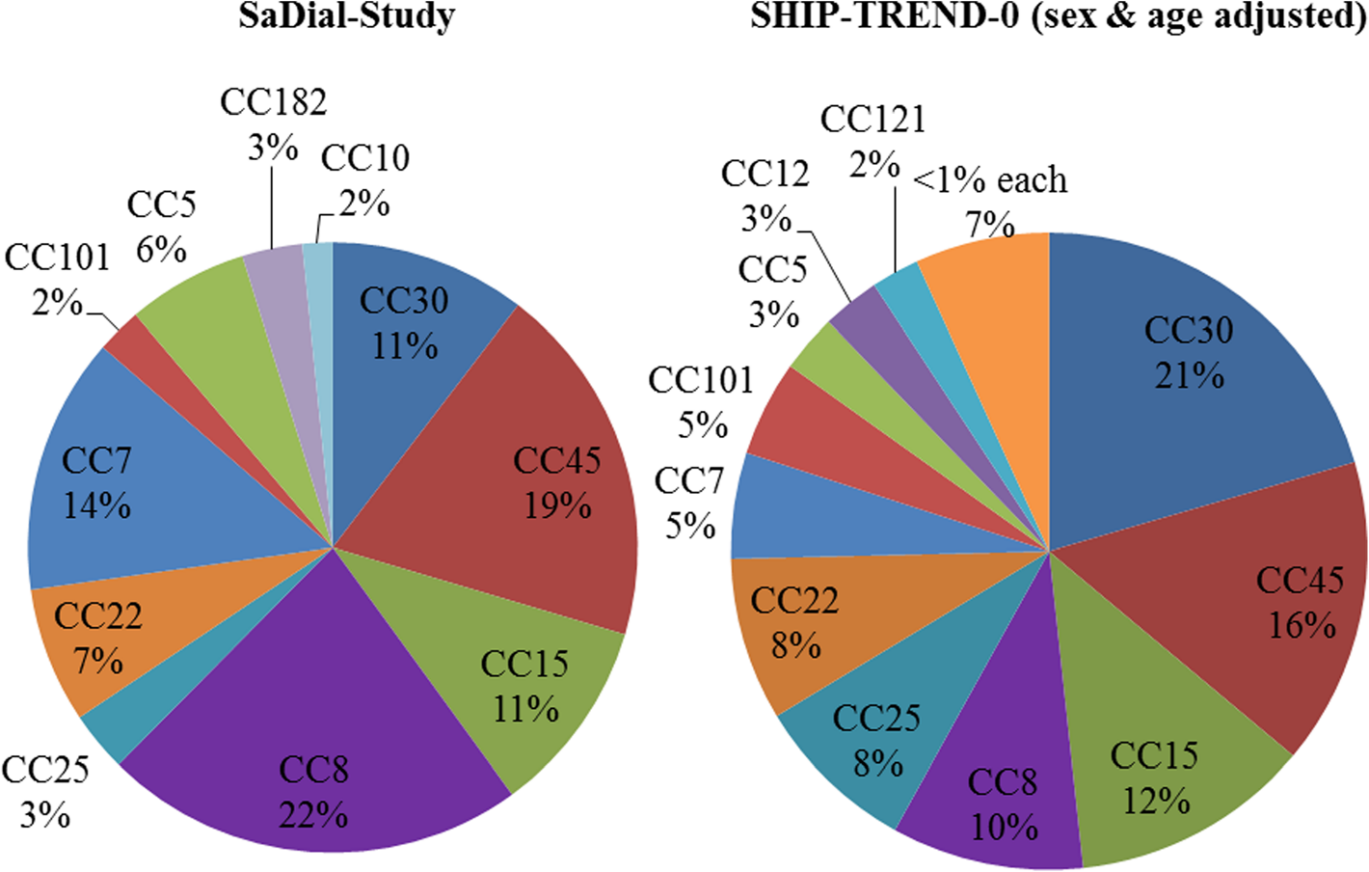
Comparison of clonal complex (CC) frequencies in healthy population (SHIP-TREND-0) with dialysis patients (SaDial-study). Clonal complexes 7 and 8 are more than twice as common in dialysis patients as in the healthy population. In contrast, the CC30 is represented only half as often

The colonizing *S. aureus* population in hemodialysis patients was very diverse. The most common sequence type was the CC8 (22%) followed by CC45 (19%) and CC7 (14%) while the CC30 (11%) was only placed on position four (Fig. 3). The two positive MRSA nasal swabs were assigned to the sequence types CC5 and CC22. These, as well as the clonal complexes CC1, CC10, CC25, CC101, CC133 and CC182 could also be detected sporadically (Table 2). This distribution differs from the sex- and age-adjusted SHIP-TREND-0 general population (*p* = 0.001). The most common *S. aureus* strain here was the CC30 (21%) (twice as frequent compared to SaDial) followed by CC45 (16%), CC15 (12%), CC8 (10%) (half as frequent), CC25 (8%), CC22 (8%) and CC7 (5%) (third as frequent). All other sequence types matched in relative numbers in both studies.

**Table 2:** Prevalence of *S. aureus* lineages (CCs) among dialysis patients during five biannual samplings

MLST-based clonal complex	Feb 2016		Aug 2016		Feb 2017		Sep 2017		Feb 2018		
	No	%	No	%	No	%	No	%	No	%	Total
CC5	1	3.4	2	8.7	2	7.1	2	8.7	1	4.0	8
CC7	2	6.9	3	13.0	5	17.9	3	13.0	4	16.0	17
CC8	8	27.6	5	21.7	6	21.4	5	21.7	4	16.0	28
(t008	8	27.6	4	17.4	4	14.3	5	21.7	4	16.0	25)
CC15	5	17.2	2	8.7	4	14.3	1	4.3	1	4.0	13
CC22	2	6.9	2	8.7	2	7.1	1	4.3	2	8.0	9
CC30	4	13.8	3	13.0	1	3.6	3	13.0	2	8.0	13
CC45	6	20.7	3	13.0	4	14.3	4	17.4	7	28.0	24
Further types	CC25		CC25, CC101, CC182		CC10, CC25, CC101, CC182		CC25, CC101, CC133, CC182		CC12, CC133, CC182, CC1		16

A closer look at the *spa* types showed that the *spa* type t008 was highly prevalent in the dialysis cohort (19%, considering all positive samples) (Table 2). A comparison of all t008 *spa* types on genetic level by DNA microarray revealed that these isolates were not clonally identical. All other *spa* types were only occasionally detected.

We frequently detected one or more strain shifts in persistent carriers (9/14) (Additional file 1). In 24 cases, the primary and the subsequent isolate were closely related based on their *spa* type, suggesting the presence of a diversifying *S. aureus* population in the nose rather than a strain replacement. In 11 cases, the subsequent isolate belonged to a different *spa* type, implying a strain replacement. However, since only one colony from each patient was analyzed, we cannot exclude a nasal co-colonization with different *S. aureus* clones. Patient 005, for instance, was co-colonized with two different *spa* types (t593 (CC15) and t091 (CC7)) in February 2016 with one of the types also being detected at the CVC entry site.

### *S. aureus* infections

Contrary to expectations, there were only six blood stream infections with *S. aureus* in the dialysis cohort within the 25 sampling months (Table 3). Affected patients showed the corresponding symptoms (leukocytosis, fever and systemic inflammatory response syndrome) and were tested positive for *S. aureus* in blood culture. Three of these infections were attributed to the CVC access. All patients but one survived the *S. aureus-*associated infection. There was no link between the infection and the *S. aureus* carrier status or the type of dialysis access. Four of these six patients were dialyzed by a CVC at the time of infection (ID 005, 018, 019, 028), while two patients were dialyzed via AV fistula continuously over all sampling months (ID 032, 034). This corresponds to a rate of 0.002 *S. aureus* infections per month for AF patients and 0.01 for CVC patients respectively.

**Table 3:** Overview of all 6 *S. aureus*-related infections

		S. aureus isolate at sampling					Blood culture		Infection	Sur-vived?
Patient-ID^a^	Loc.^b^	Feb 2016	Aug 2016	Feb 2017	Sep 2017	Feb 2018	Time-point	Isolate		
005	Nose	t593/CC15	t593/CC15	t091/CC7	t2636/CC15	deceased	Sep 16	t593/CC15	Endogenous	Yes
		t091/CC7	t091/CC7							
	CVC	t593/CC15	no	t2636/CC15	t091/CC7					
018	Nose	negative					Jun 16	t232/CC101	Exogenous	Yes
	CVC	negative			no CVC					
019	Nose	negative			deceased		Jun 17	Unknown	Exogenous	No
	CVC									
028	Nose	not yet participated		t16794/CC7	negative		Aug 16	t16794/CC7	Unknown	Yes
	CVC			negative	no CVC					
032	Nose	t548/CC5	negative	t548/CC5	t548/CC5	t548/CC5	Aug 16	t056/CC101	Exogenous	Yes
	CVC	no CVC								
034	Nose	t008/CC8	negative		withdrawal		May 16	t008/CC8	Endogenous	Yes
	CVC	no CVC								

^a^Table is read line by line for each patient ID. Each patient ID is divided into detections in nose and CVC for each sampling month. Same results are framed

^2^Loc. – swab location (Nose and if present CVC); negative – no *S. aureus* bfound

To investigate whether the infection was of endogenous or exogenous origin, we performed spa typing of the invasive isolates. In two patients the invasive isolate had the same *spa* type as the nasal isolate (obtained a month earlier (ID 005) or three months earlier (ID 034), implying an endogenous infection. Another two patients were non-carriers who acquired an exogenous infection (ID 018, 019). Moreover, one carrier was infected by an unrelated, exogenous strain (ID 032).

### Causes for overall mortality of dialysis patients

During the 25-months study period 22/86 patients died (mortality: 26%). Main cause of death was a sepsis or septic shock (16/22 patients), but only in one case (ID 019) *S. aureus* could be confirmed as the causative pathogen (Table 4). This patient was a non-carrier during all samplings and died due to a *S. aureus*-induced infection of the central venous catheter with further involvement of gram-negative bacteria (Table 4). Seven septic patients had no clear evidence of *S. aureus* because the focus remained unclear or could only be limited to cocci in general. In another 8 septic patients, sepsis was induced by other pathogens (*E. faecalis*, *S. epidermidis*, *E. coli*, *C. difficile, K. oxytoca, P. aeruginosa and Influenza B*). Only one patient died from sepsis due to a proven CVC infection caused by *Klebsiella oxytoca*. Other five patients had a suspected CVC infection without pathogen detection. Only 6 patients overall did not die from sepsis but from either ischemic or embolic diseases (Table 4).

**Table 4:** Overview of all 22 deceased patients including causes of death and responsible pathogens

Focus	Cause of death	Pathogen detection	SaDial-ID
Septic	Sepsis (mesenteric ischemia)	*S. aureus*, *E. faecalis*	019
	Septic shock (diabetic foot gangrene)	*S. epidermidis*	011
	Sepsis (focus unknown, either pneumonia or CVC)	no	013
	Sepsis (CVC infection)	no	014
	Sepsis (aspiration pneumonia)	*E. coli*	021
	Sepsis (gangrenous cholecystitis)	*Cocci*	023
	Sepsis (pneumonia)	*Enterococci*	033
	Sepsis (focus unknown, either pneumonia or CVC)	no	080
	Septic shock (unknown focus, possible CVC sepsis)	no	001
	Sepsis (infectious colitis)	*C. difficile*	053
	Recurrent sepsis by various pathogens (metastatic cholangiocellular carcinoma)	inter alia *C. difficile*	101
	Sepsis (unknown focus, possible CVC sepsis)	gram-negative *Cocci*, *K. oxytoca*	005
	Sepsis (pneumonia)	*Influenza B*	034
	Sepsis and congestive heart failure (possibly endocarditis after transcatheter aortic valve implantation)	*E. faecalis*	056
	Respiratory failure, chronic emphysema and recurrent infections	*P. aeruginosa* in sputum	072
	Septic shock (unknown focus, possible CVC sepsis)	no	010
Cardio-logical	Congestive heart failure/ cardiogenic shock	no	022
	Congestive heart failure/ cardiogenic shock	no	015
	Hypoxic brain damage after resuscitation in ventricular fibrillation	no	003
Intestinal	Non-occlusive mesenteric ischemia	no	016
	Intestinal perforation	no	068
Pulmonary	Pulmonary embolism	no	039

To investigate the impact of demographic data and patients´ medical histories from our dialysis cohort, we performed a multivariate analysis (Cox regression). As expected, advanced age was an independent factor positively associated with mortality (*p* = 0.005). Unexpectedly, *S. aureus* carriers had a 5-fold higher survival probability compared to non-carriers (HR, 0.2 for carriers to die [95% CI, 0.1–0.6]). Overall mortality considering the sub-cohort of *S. aureus* carriers was in turn only significantly influenced by patient age.

**Fig. 4:**
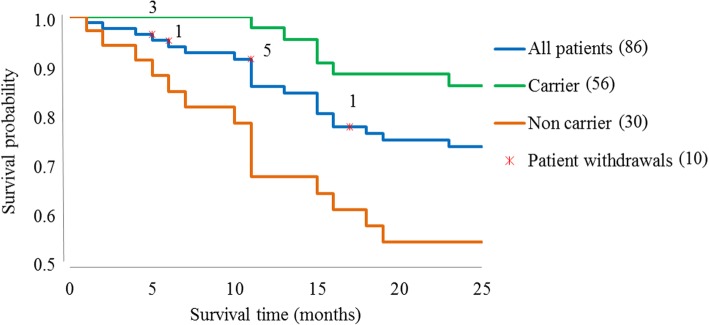
Kaplan-Meier survival curve of dialysis patients over 25 months. The survival rate over all patients after the last sampling was 74%. The survival probability for the sub-cohort *S. aureus* non-carrier was about 20% lower and 12% higher for *S. aureus* carrier. Number of patients considered in brackets

The survival probability in the hemodialysis study population over 25 months was 74% with a standard deviation of 3%. Figure 4 displays the Kaplan-Meier survival curves of all dialysis patients as well as for the sub-cohorts *S. aureus* carrier and non-carrier. In patients with at least one *S. aureus* positive nasal swab during all samplings, the overall survival probability raised to 86%. In contrast, the survival probability diminished to 55% in non-carriers.

## Discussion

Cross-sectionally, between 33 and 46% of the studied hemodialysis patients were *S. aureus* carriers. However, longitudinally over 25 months, we found 65% *S. aureus* carriers: 43% intermittent, 16% persistent carriers and 6% with only one *S. aureus* detection without further sampling option. Only two patients were intermittently colonized with MRSA (CC5, CC22). The most common *S. aureus* strain in the dialysis patient cohort was the clonal complex CC8 and the *spa* type t008, while in general population of the same geographical region CC30 dominates. The *S. aureus* colonization density in patients with an AV fistula was significantly higher compared to those dialyzed via CVC possibly explained by longer dialysis dependency (> 3 years). During follow-up, only six out of 86 patients developed *S. aureus* induced blood stream infections: two with an endogenous, three with an exogenous origin and one remained unclear. A positive *S. aureus* carrier status was associated with a lower overall-mortality.

### Low *S. aureus* prevalence in SaDial study on hemodialysis patients

The SaDial study revealed a cross-sectional *S. aureus* prevalence in hemodialysis patients between 33 and 46%, which is higher than the local general population (SHIP-TREND-0, mean 25%), but rather in the lower area compared to previous studies on hemodialysis patients (ranging from 30 to 84%) [8]. The strong variations in the carrier rate between different study cohorts are still common today and are likely influenced by the type of dialysis [17]. Among *S. aureus* carriers in our study, hemodialysis patients with an AV fistula had been on renal replacement therapy for longer time than those with CVC and had a higher *S. aureus* density at nasal swabs. Usually, patients with an AV fistula are those whose renal disease has a longer history. Thus, they had more chances to come into contact with hospital environment, medical staff and also with *S. aureus*. This way, both the likelihood of colonization and the colonization density increase.

The MRSA prevalence in the SaDial hemodialysis patients (2/86 patients; 2.3%) was well below the average MRSA prevalence as summarized in a meta-analysis of > 5.000 hemodialysis patients worldwide (7.2%, range 4.9–9.9%; [18]). Related to the same geographical region, the MRSA prevalence of the SaDial-study is above the normal population (0.34%) [3] and slightly above German health care facilities of northeastern Germany: 2.1% in homecare services, 1.4% in long-term care facilities, and 0.5% in rehabilitation clinics [19]. A comparative study by Neuhaus et al. in 2003 considering the MRSA prevalence in German hospitals and nursing homes showed no significant difference between both medical institutions in case of an active exchange of patients [20]. Similar to our outpatient dialysis center and the University Medicine Greifswald, this leads to an adjustment of MRSA prevalence to the latter one. However, established prevention strategies against MRSA spread, such as information exchange to the patient’s carrier status, the subsequent isolation of affected ones or eventual decolonization actions of MRSA carriers, prevent a spread of MRSA strains and show in a worldwide comparison a low MRSA prevalence in our outpatient dialysis center.

### Different *S. aureus* properties in hemodialysis patients

We compared the *S. aureus* population from our study cohort with data from a population-based study of the same geographical region of Western Pomerania (Study of Health in Pomerania, SHIP-TREND-0). The study investigated 3891 subjects aged between 29 and 79 and tested for nasal colonization with *S. aureus*. We adjusted the data according to sex and age. Clonal complexes occurring in patients requiring hemodialysis differ from those found in the general population with CC7 and CC8 being more prevalent in the patient cohort and CC30 being underrepresented (*p* = 0.001). In contrast, a study of dialysis patients from England in 2002 revealed a distribution of CC types corresponding rather to the general population from the SHIP-TREND-0 study [3]. The CC30 was the most prevalent one, followed by CC25, CC22 and CC8. Only the MLST type CC45 was underrepresented [21]. The different MLST distributions of SaDial and SHIP-TREND-0 are in agreement with the general observation that *S. aureus* colonization characteristics differ from the general population if patients are in frequent contact with hospitals, dialysis centers or homes for the elderly.

**Fig. 5:**
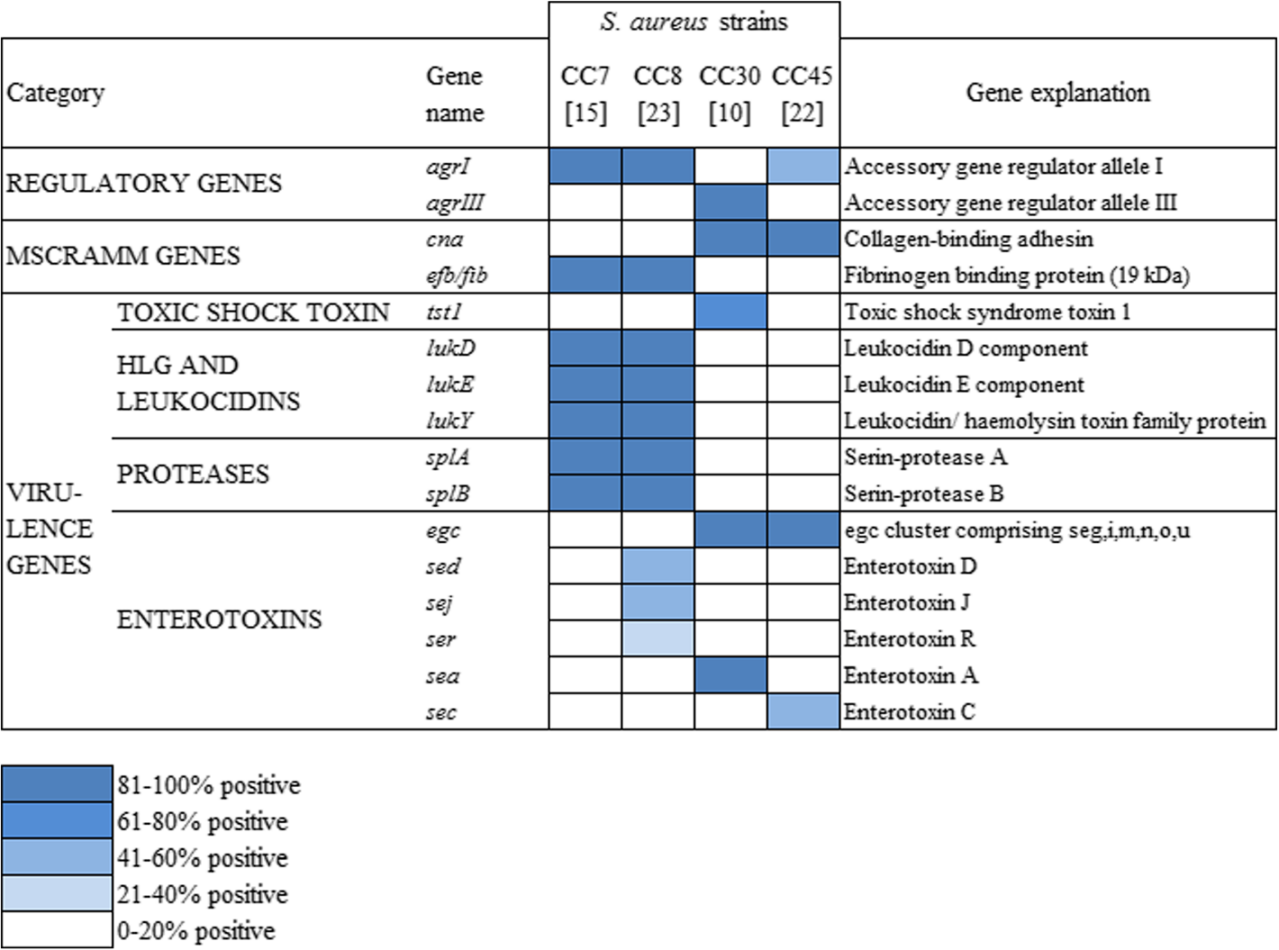
Comparison of existing genes in clonal complexes (CC) CC7, CC8, CC30 and CC45 of *S. aureus*. CC7 and CC8 show great similarities in regulatory and virulence genes as well in genes for microbial surface components recognizing adhesive matrix molecules (MSCRAMM) and differ from CC30 and CC45. Number of strains considered for each clonal complex in square brackets

Each *S. aureus* lineage is equipped with a specific set of surface-attached adhesins, as well as toxins and immune evasion molecules; we tested whether CC7 and CC8 share some properties that might facilitate transmission and colonization in a hemodialysis setting, that are lacking in CC30 (Fig. 5). Interestingly, both CC7 and CC8 harbor the fibrinogen binding protein (*efb/fib*), which contributes to the initiation of foreign body infections [22]. In contrast, the collagen binding adhesion (*can*) mediating bacterial adherence to collagen substrates and collagenous tissues [23] is not expressed in CC8 and CC7 but in CC30. These genetic differences illustrate the strong influence of the medical environment on the *S. aureus* population and in consequence on the distribution of lineage-associated virulence factors.

### Mortality data and *S. aureus* carrier status

After 24 months, the mortality rate in the hemodialysis study population was 26% (Fig. 3), with the overall mortality being lower for *S. aureus* carriers than non-carriers (HR 0.19, *p* = 0.005). The overall mortality is comparable to data from literature [24, 25]. However, the fact that *S. aureus* carriers seem to be protected needs to be studied more closely.

Protection from invasive *S. aureus* infections could be explained by the stronger pre-existing immune memory in carriers. In 2005, Wertheim et al. already showed that *S. aureus* carriers may have a decreased risk of death compared to non-carriers in case of *S. aureus* infection, hypothesizing that anti-staphylococcal antibody levels are increased in carriers, induced by prior *S. aureus* exposure and playing a role in protection [4]. By now, it is well known that anti- *S. aureus* antibody titers in carriers are higher than in non-carriers [26]. In 2015, van den Berg could strengthen this hypothesis in a mouse model by improving the course of subsequent endogenous *S. aureus* bacteremia by mild *S. aureus* skin infections. *S. aureus* skin infected mice showed IgG levels against various *S. aureus* antigens and had a higher survival probability [27]. Moreover, immune memory of *S. aureus* was associated with protection from serious complications of bacterial invasion [28]. The mortality rate of *S. aureus* carriers in our study population was 32% lower compared to non-carriers after 25 months. In conclusion, *S. aureus* colonization along with subclinical infections may prime the adaptive immune system and lead to a more robust immune memory protecting from fatal *S. aureus* sepsis.

Nevertheless, only one death in the SaDial study could clearly be attributed to a *S. aureus* sepsis. Indeed, the main causes of death in our hemodialysis study population were bloodstream infections by pathogens other than *S. aureus* (73%), followed by cardiovascular events (14%), and various causes (13%). Previous studies report that the main causes of death are comorbidities that occur alongside with or result from renal insufficiency [24]: cardiovascular events (40%), bloodstream infections (30%) and sudden deaths of patients at home with unknown cause (25%) [29, 30], the latter often again being attributed to cardiovascular events [28]. How *S. aureus* carriage could influence the overall mortality due to non-*S. aureus* infections and other, non-infectious diseases remains to be clarified.

Contrary to our assumption that *S. aureus* was the main cause of blood stream infections, mainly other pathogens such as *E. faecalis, S. epidermidis*, *E. coli*, *C. difficile K. oxytoca* and *Influenza B* were isolated from blood culture. According to a nationwide study in the USA by Wisplinghoff et al. in 2004 considering 24,000 infections from 10,000 hospitals, 27% were caused by coagulase negative staphylococci, 24% by *S. aureus*, 9% by *Enterococcus species*, 8% by *Candida species*, 8% by *E. coli*, 6% by *Klebsiella species* and others [31]. Mortality rate varied according to the respective bacteria between 13 and 29% (*Candida species* 29%, *Enterococcus species* 24%, *S. aureus* 19%, *E.coli* 17%) [31]. Thus, pathogens detected in our patients are among the common causes of blood stream infections. Future studies will use serodiagnostics, i.e. the induction of a species-specific antibody response, to clarify which pathogens were recognized and combated by the adaptive immune system. Hopefully, this will shed some light on the physiologically relevant invasive pathogens that might have been missed by standard microbiological diagnosis.

## Conclusion

Sixty five percent of hemodialysis patients in our study population were either intermittent or persistent *S. aureus* carriers with high variability in *S. aureus* colonization. Overall, our study population showed a comparatively low colonization rate with *S. aureus* and a lower blood stream infection rate with *S. aureus* than expected. Compared to the general population, we found a different distribution of *S. aureus* strains in our hemodialysis patients. Furthermore, overall mortality was lower for hemodialysis patients with a positive *S. aureus* carrier status. Regular and frequent contact with professional health care givers may explain both the observed colonization shift as well as the difference in colonization density. Contact with *S. aureus* may lead to a stronger immune memory of *S. aureus* and may protect the patient in case of infection.

## Additional file

Additional file 1: Overview on detected *S. aureus* isolates and their classification. ^1^Table is read line by line for each patient ID. Each patient ID is divided into detections in nose and CVC for each sampling month. Same results are framed. ^2^Loc. – swab location (Nose and if present CVC); negative – no *S. aureus* found; no – no CVC existing; MRSA in bold. (XLSX 23 kb)

## Abbreviations

AV: Arteriovenous
CC: Clonal complex
CVC: Central venous catheter
ICU: Intensive care unit
MLST: Multilocus sequence typing
MRSA: Methicillin-resistant *Staphylococcus aureus*
MSCRAMM: Microbial surface components recognizing adhesive matrix molecules
MSSA: Methicillin-susceptible *S. aureus*

## References

[1] Mulcahy ME, McLoughlin RM (2016). Host–bacterial crosstalk determines *Staphylococcus aureus* nasal colonization. Trends Microbiol.

[2] den Heijer CDJ, van Bijnen EME, Paget WJ, Pringle M, Goossens H, Bruggeman CA, Schellevis FG, Stobberingh EE (2013). Prevalence and resistance of commensal Staphylococcus aureus, including meticillin-resistant *S aureus*, in nine European countries: a cross-sectional study. Lancet Infect Dis.

[3] Holtfreter S, Grumann D, Balau V, Barwich A, Kolata J, Goehler A, Weiss S, Holtfreter B, Bauerfeind SS, Döring P, Friebe E, Haasler N, Henselin K, Kühn K, Nowotny S, Radke D, Schulz K, Schulz SR, Trübe P, Vu CH, Walther B, Westphal S, Cuny C, Witte W, Völzke H, Grabe HJ, Kocher T, Steinmetz I, Bröker BM (2016). Molecular epidemiology of *Staphylococcus aureus* in the general population in Northeast Germany: results of the study of health in Pomerania (SHIP-TREND-0). J Clin Microbiol.

[4] Wertheim HF, Melles DC, Vos MC, van Leeuwen W, van Belkum A, Verbrugh HA, Nouwen JL (2005). The role of nasal carriage in *Staphylococcus aureus* infections. Lancet Infect Dis.

[5] Fowler VG, Olsen MK, Corey GR, Woods CW, Cabell CH, Reller LB, Cheng AC, Dudley T, Oddone EZ (2003). Clinical identifiers of complicated *Staphylococcus aureus* bacteremia. Arch Intern Med.

[6] Chang FY, MacDonald BB, Peacock JE, Musher DM, Triplett P, Mylotte JM, O'Donnell A, Wagener MM, Yu VL (2003). A prospective multicenter study of *Staphylococcus aureus* bacteremia: incidence of endocarditis, risk factors for mortality, and clinical impact of methicillin resistance. Medicine (Baltimore).

[7] von Eiff C, Becker K, Machka K, Stammer H, Peters G (2001). Nasal carriage as a source of *Staphylococcus aureus* bacteremia. Study Group. N Engl J Med.

[8] Kluytmans J, van Belkum A, Verbrugh H (1997). Nasal carriage of *Staphylococcus aureus*: epidemiology, underlying mechanisms, and associated risks. Clin Microbiol Rev.

[9] Astor BC, Eustace JA, Powe NR, Klag MJ, Fink NE, Coresh J (2005). Type of vascular access and survival among incident hemodialysis patients: the choices for healthy outcomes in caring for ESRD (CHOICE) study. J Am Soc Nephrol.

[10] Lafrance JP, Rahme E, Lelorier J, Iqbal S (2008). Vascular access-related infections: definitions, incidence rates, and risk factors. Am J Kidney Dis.

[11] Sarnak MJ, Jaber BL (2000). Mortality caused by sepsis in patients with end-stage renal disease compared with the general population. Kidney Int.

[12] Layer F, Strommenger B, Cuny C, Werner G (2018). Eigenschaften, Häufigkeit und Verbreitung von MRSA in Deutschland. Epid Bull.

[13] Aghdassi S, Behnke M, Gastmeier P, Gropmann A, Hansen S, Diaz LA, Piening B, Rosenbusch M, Schröder C, Schwab F (2016). Deutsche nationale Punkt-Prävalenzerhebung zu nosokomialen Infektionen und Antibiotika-Anwendung 2016: Abschlussbericht. Nationales Referenzzentrum für Surveillance von nosokomialen Infektionen.

[14] Köck R, Mellmann A, Schaumburg F, Friedrich AW, Kipp F, Becker K (2011). Methicillin-resistenter *Staphylococcus aureus* in Deutschland. Dtsch Arztebl Int.

[15] Shopsin B, Gomez M, Montgomery SO, Smith DH, Waddington M, Dodge DE, Bost DA, Riehman M, Naidich S, Kreiswirth BN (1999). Evaluation of protein a gene polymorphic region DNA sequencing for typing of *Staphylococcus aureus* strains. J Clin Microbiol.

[16] Monecke S, Slickers P, Ehricht R (2008). Assignment of Staphylococcus aureus isolates to clonal complexes based on microarray analysis and pattern recognition. FEMS Immunol Med Microbiol.

[17] Price A, Sarween N, Gupta I, Baharani J (2015). Meticillin-resistant Staphylococcus aureus and meticillin-susceptible *Staphylococcus aureus* screening in a cohort of haemodialysis patients: carriage, demographics and outcomes. J Hosp Infect.

[18] Zacharioudakis IM, Zervou FN, Ziakas PD, Mylonakis E (2014). Meta-analysis of methicillin-resistant *Staphylococcus aureus* colonization and risk of infection in Dialysis patients. J Am Soc Nephrol.

[19] Hübner NO, Dittmann K, Begunk R, Kramer A (2017). Infection control measures and prevalence of multidrug-resistant organisms in non-hospital care settings in northeastern Germany: results from a one-day point prevalence study. J Hosp Infect.

[20] Neuhaus B, Bocter N, Braulke C, Heuck D, Witte W. Methicillin-resistente Staphylokokken: In Altenheimen ebenso häufig vertreten wie in Krankenhäusern. Dtsch Arztebl. 2003;100(45).

[21] Peacock SJ, de Silva GDI, Justice A, Cowland A, Moore CE, Winearls CG, Day NPJ (2002). Comparison of multilocus sequence typing and pulsed-field gel electrophoresis as tools for typing *Staphylococcus aureus* isolates in a microepidemiological setting. J Clin Microbiol.

[22] Herman-Bausier P, El-Kirat-Chatel S, Foster TJ, Geoghegan JA, Dufrêne YF (2015). *Staphylococcus aureus* fibronectin-binding protein a mediates cell-cell adhesion through low-affinity Homophilic bonds. MBio..

[23] Switalski LM, Patti JM, Butcher W, Gristina AG, Speziale P, Hook M (1993). A collagen receptor on *Staphylococcus aureus* strains isolated from patients with septic arthritis mediates adhesion to cartilage. Mol Microbiol.

[24] Lin Y, Wu P, Kuo M, Lin M, Lee T, Chiu Y, Hwang S, Chen HC (2013). High cost and low survival rate in high comorbidity incident elderly hemodialysis patients. PLoS One.

[25] Foster BJ, Mitsnefes MM, Dahhou M, Zhang X, Laskin BL (2018). Changes in excess mortality from end stage renal disease in the United States from 1995 to 2013. Clin J Am Soc Nephrol.

[26] Bröker BM, Holtfreter S, Bekeredjian-Ding I (2014). Immune control of *Staphylococcus aureus* - regulation and counter-regulation of the adaptive immune response. Int J Med Microbiol.

[27] van den Berg S, de Vogel CP, van Belkum A, Bakker-Woudenberg IAJM (2015). Mild *Staphylococcus aureus* skin infection improves the course of subsequent Endogenous S. aureus bacteremia in mice. PLoS One.

[28] Stentzel S, Sundaramoorthy N, Michalik S, Nordengrün M, Schulz S, Kolata J, Kloppot P, Engelmann S, Steil L, Hecker M, Schmidt F, Völker U, Roghmann M-C, Bröker BM (2015). Specific serum IgG at diagnosis of *Staphylococcus aureus* bloodstream invasion is correlated with disease progression. J Proteome.

[29] Zamiah SS, Draman CR, Seman MR, Safhan AF, Rozalina R, Nik Ruzni NI (2018). The cardiovascular risk factor profiles among end-stage renal failure patients treated with continuous ambulatory peritoneal dialysis and intermittent hemodialysis. Saudi J Kidney Dis Transpl.

[30] Wetmore JB, Molony JT, Liu J, Peng Y, Herzog CA, Collins AJ, Gilbertson DT (2018). Readmissions following a hospitalization for cardiovascular events in Dialysis patients: a retrospective cohort study. J Am Heart Assoc.

[31] Wisplinghoff H, Bischoff T, Tallent SM, Seifert H, Wenzel RP, Edmond MB (2004). Nosocomial bloodstream infections in US hospitals: analysis of 24,179 cases from a prospective Nationwide surveillance study. Clin Infect Dis.

